# Crystal structure and Hirshfeld surface analyses, inter­action energy calculations and energy frameworks of methyl 2-[(4-cyano­phen­yl)meth­oxy]quinoline-4-carboxyl­ate

**DOI:** 10.1107/S2056989025005547

**Published:** 2025-06-27

**Authors:** Ayoub El-Mrabet, Amal Haoudi, Frederic Capet, Tuncer Hökelek, Mazzah Ahmed

**Affiliations:** a Laboratory of Applied Organic Chemistry, Sidi Mohamed Ben Abdellah University, Faculty Of Science And Technology, Road Immouzer, BP 2202 Fez, Morocco; b University of Lille, CNRS, UMR 8181, UCCS, Unité de Catalyse et Chimie du Solide, F-59000 Lille, France; c Department of Physics, Hacettepe University, 06800 Beytepe, Ankara, Türkiye; d Université de Lille, CNRS, UAR 3290, MSAP, Miniaturization for Synthesis, Analysis and Proteomics, F-59000 Lille, France; National Taras Shevchenko University of Kyiv, Ukraine

**Keywords:** quinoline, π-stacking, weak hydrogen bond, tetrel bond, crystal structure

## Abstract

The title compound, C_19_H_14_N_2_O_3_, features competition and inter­play of a range of weak inter­actions, which actualize under the absence of conventional hydrogen-bond donors. Two kinds of stacking inter­actions, namely slipped anti­parallel inter­actions of cyano­phenyl groups as well as quinoline and carb­oxy groups, are primarily important. In combination with relatively short tetrel OCH_3_⋯N≡C bonds [C⋯N = 3.146 (3) Å] they are responsible for the generation of the layers, while the inter­layer bonding occurs *via* C—H⋯O and C—H⋯N weak hydrogen bonds. These findings are consistent with the results of Hirshfeld surface analysis and calculated inter­action energies. Contributions of the C⋯C, C⋯N/N⋯C and C⋯O/O⋯C contacts originating in the stacking inter­actions account for 17.0% to the surface area. The largest inter­actions energies are associated with the two kinds of stacks (−45.8 and −24.3 kJ mol^−1^) and they are superior to the energies of weak hydrogen bond and tetrel inter­actions (−12.4 to −22.4 kJ mol^−1^). Evaluation of the electrostatic, dispersion and total energy frameworks indicate that the consolidation is dominated *via* the dispersion energy contributions.

## Abstract 2

The crystal structure of new quinoline-4-carboxyl­ate derivative exhibits a range of weak inter­actions, which were assessed using Hirshfeld surface analysis and inter­action energy calculations to support the dominant significance of the dispersion forces.

## 1. Chemical context

Heterocyclic compounds, especially nitro­gen-containing systems such as quinoline derivatives, play a pivotal role in medicinal chemistry due to their broad spectrum of biological activities (Filali Baba *et al.*, 2019 ▸, 2020 ▸; Hayani *et al.*, 2021*a* ▸; El-Mrabet *et al.*, 2023 ▸, 2025 ▸; Bouzian *et al.*, 2018 ▸, 2021 ▸). These compounds exhibit anti­microbial (Salam *et al.*, 2023 ▸), anti­fungal (Chen *et al.*, 2021 ▸), anti-Alzheimer’s (Chen *et al.*, 2023 ▸), anti-infective (Muruganantham *et al.*, 2004 ▸), anti­leishmanial (Chanquia *et al.*, 2019 ▸), anti-HIV (Strekowski *et al.*, 1991 ▸), anti-inflammatory (Ghanim *et al.*, 2022 ▸), anti­viral (Kaur & Kumar, 2021 ▸), and corrosion inhibitive activities (Mahamoud *et al.*, 2006 ▸; Filali Baba *et al.*, 2016*a* ▸,*b* ▸). Their structural flexibility and ability to inter­act with diverse biological targets make quinolines attractive frameworks for drug development, especially in addressing significant therapeutic challenges. In this context, we report herein the synthesis and comprehensive structural characterization of a novel quinoline-based compound, methyl 2-(4-cyano­benz­yloxy)quinoline-4-carboxyl­ate (I). The target mol­ecule was obtained *via* an O-alkyl­ation reaction of methyl 2-oxo-1,2-di­hydro­quinoline-4-carboxyl­ate with 4-(bromo­meth­yl)benzo­nitrile under phase-transfer catalysis (PTC). The synthesized compound was analyzed using ^1^H and ^13^C NMR, FT-IR spectroscopy, single-crystal X-ray diffraction, and Hirshfeld surface analysis to elucidate its mol­ecular and crystal structure.
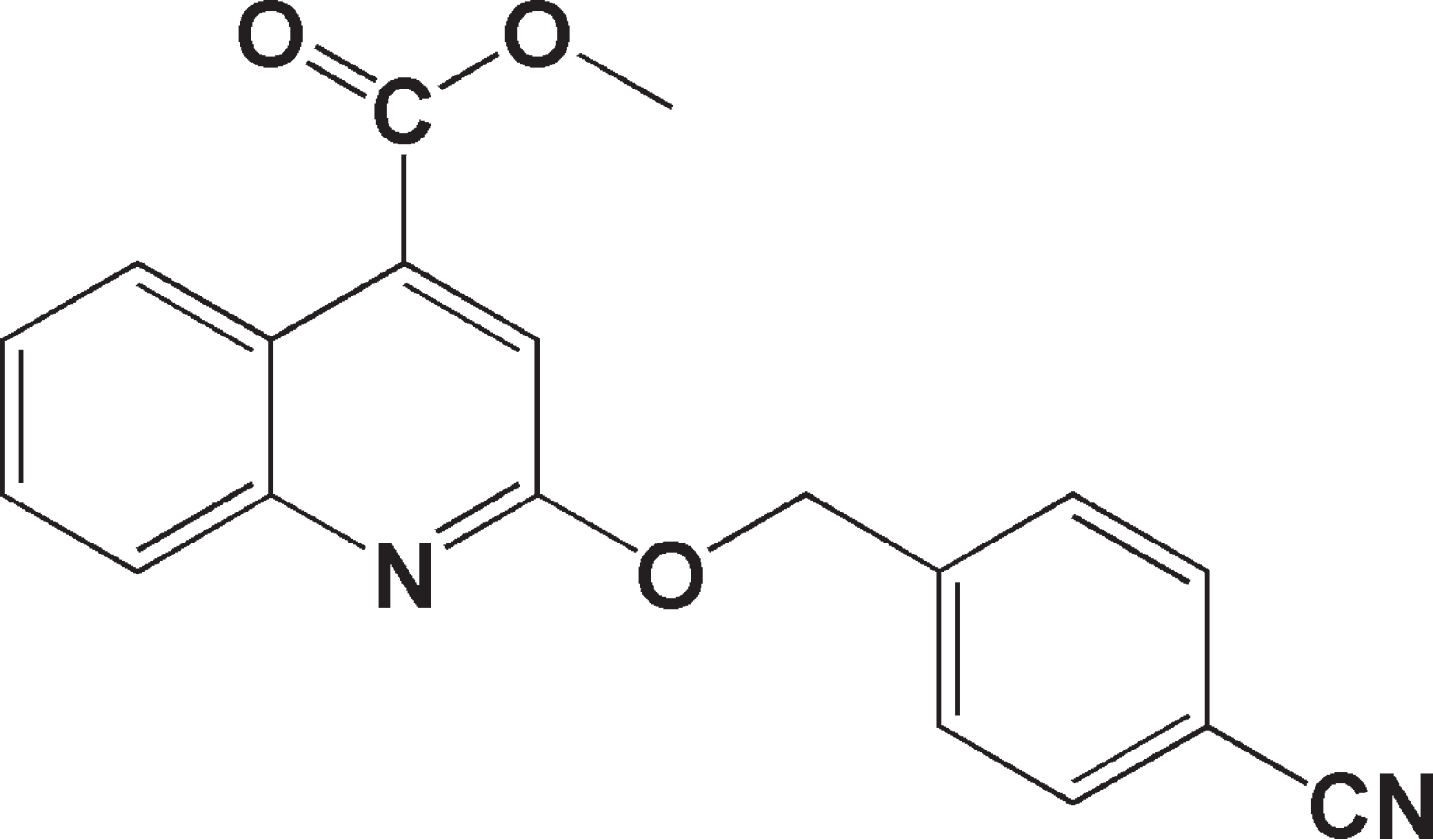


## 2. Structural commentary

**Figure 1:**
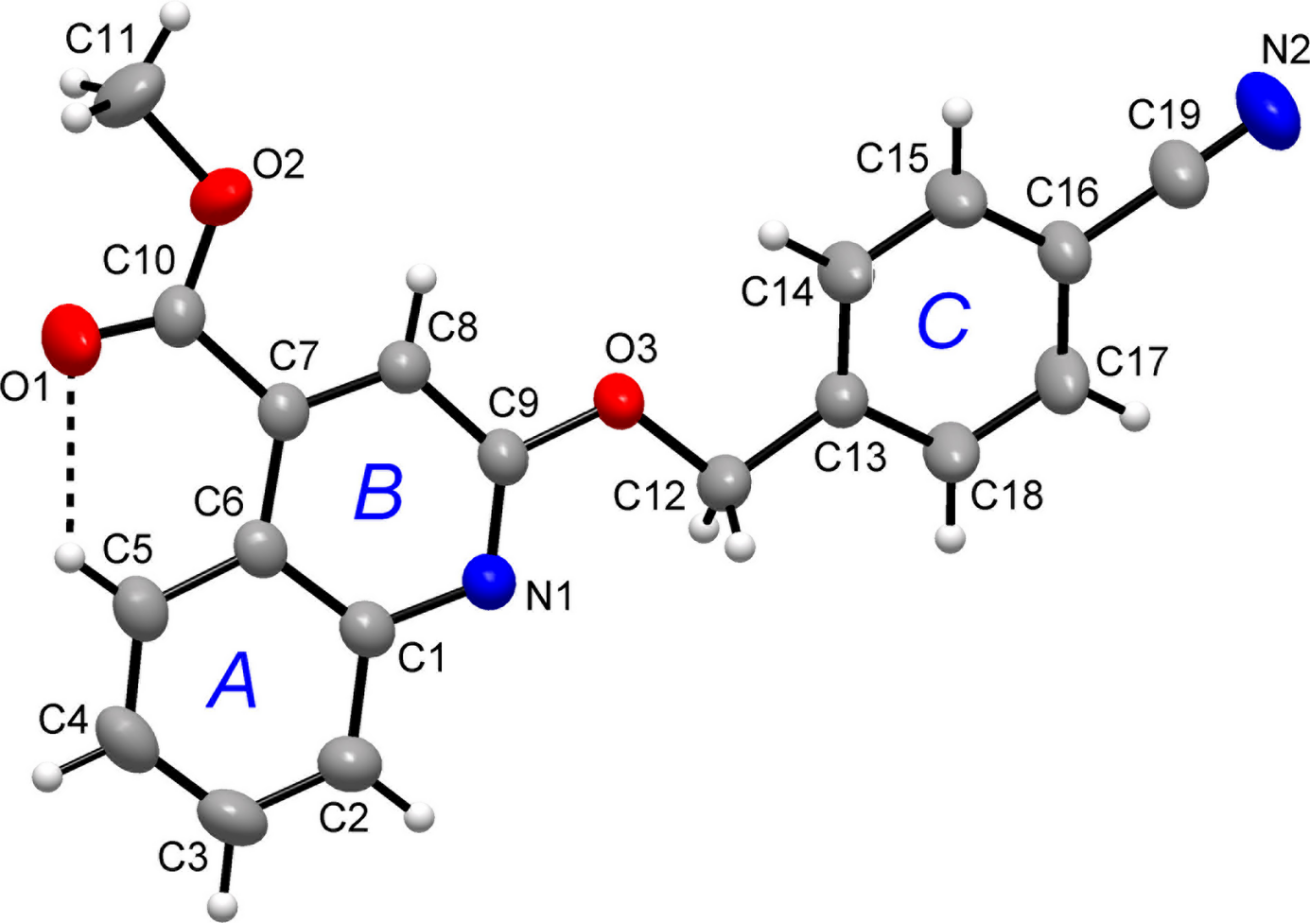
The mol­ecular structure of the title compound, with the atom and ring labelling schemes and displacement ellipsoids drawn at the 50% probability level. The dotted line indicates a possible weak hydrogen bond.

The title compound, (I), contains the almost planar quinoline and cyano­phenyl moieties (Fig. 1 ▸), where the planar *A* (C1–C6), *B* (N1/C1/C6–C9) and *C* (C13–C18) rings are oriented at dihedral angles of *A*/*B* = 0.56 (5)°, *A*/*C* = 14.47 (6)° and *B*/*C* = 15.02 (6)°. The exocyclic atoms O1, O2, O3, C10, C11 and C12 are also nearly coplanar with the quinoline framework and lie 0.005 (2), −0.030 (2), 0.016 (1), −0.015 (2), −0.067 (3) and −0.037 (2) Å, respectively, away from its mean plane.

In the ester group, the O1—C10 and O2—C10 bond lengths are 1.177 (2) Å and 1.308 (2) Å, respectively. This strict differentiation of the C—O bonds indicates mainly the localized single and double bounds rather than delocalized bonding arrangement. The O1—C10—O2 bond angle of 121.6 (2)° agrees well with the parameters for comparable methyl 2-phenyl quinoline-4-carboxyl­ate [122.42 (14)°; Mague *et al.*, 2016 ▸], and methyl 2-oxo-1-(propyn-2-yl)-1,2-di hydro­quinoline-4-carboxyl­ate [122.55 (12)°; El-Mrabet *et al.*, 2023 ▸]. The planes of the carbometh­oxy group [defined by the atoms C7, C10, O1 and O2] and ring *B* are related by 0.96 (17)° indicating a coplanar arrangement. The latter is partly caused by the weak intra­molecular C5—H5⋯O1 hydrogen bond (Table 1 ▸), similarly to in methyl 6-chloro-1-methyl-2-oxo-1,2-di­hydro­quinoline-4-carboxyl­ate with a corresponding dihedral angle of 4.08 (8)° (Filali Baba *et al.*, 2022 ▸). As evidenced by the C11—O2—C10—C7 [178.98 (18)°] torsion angle, the ester group attached to the quinoline moiety is in a *syn* peripheral conformation. The corresponding torsion angles for the related derivatives of benzyl [176.06 (11)°; Bouzian *et al.*, 2018 ▸] and ethyl [−176.71 (15)°; Sunitha *et al.*, 2015 ▸] quinoline-4-carboxyl­ates represent *syn-* and *anti-*peripheral conformations, respectively.

## 3. Supra­molecular features

**Figure 2:**
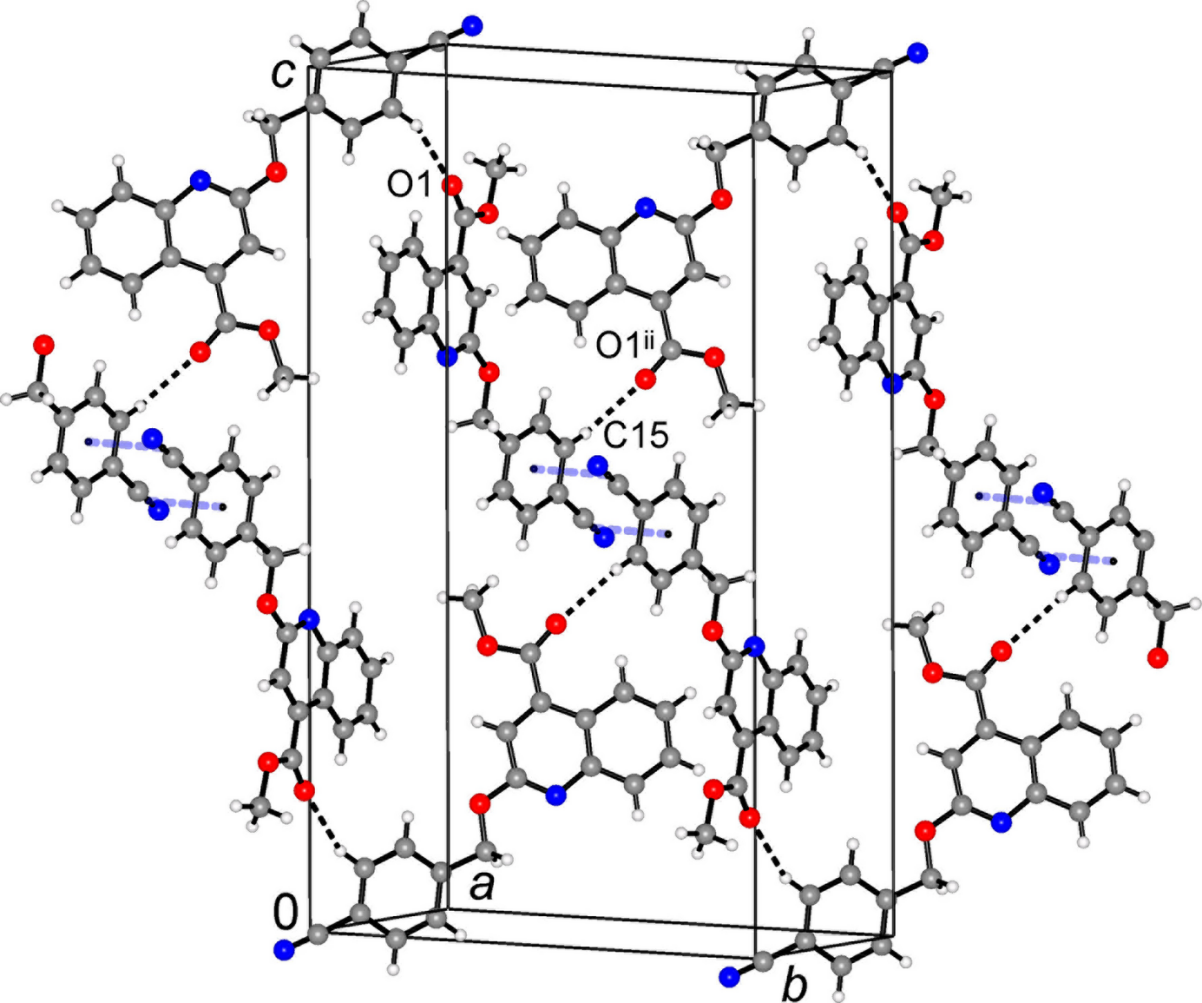
Fragment of the crystal structure showing hydrogen-bonded chains along the *b*-axis direction and stacking inter­actions between the adjacent chains. [Symmetry code (ii): *x* + 1, *y* + 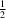, *z* + 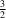.]

**Figure 3:**
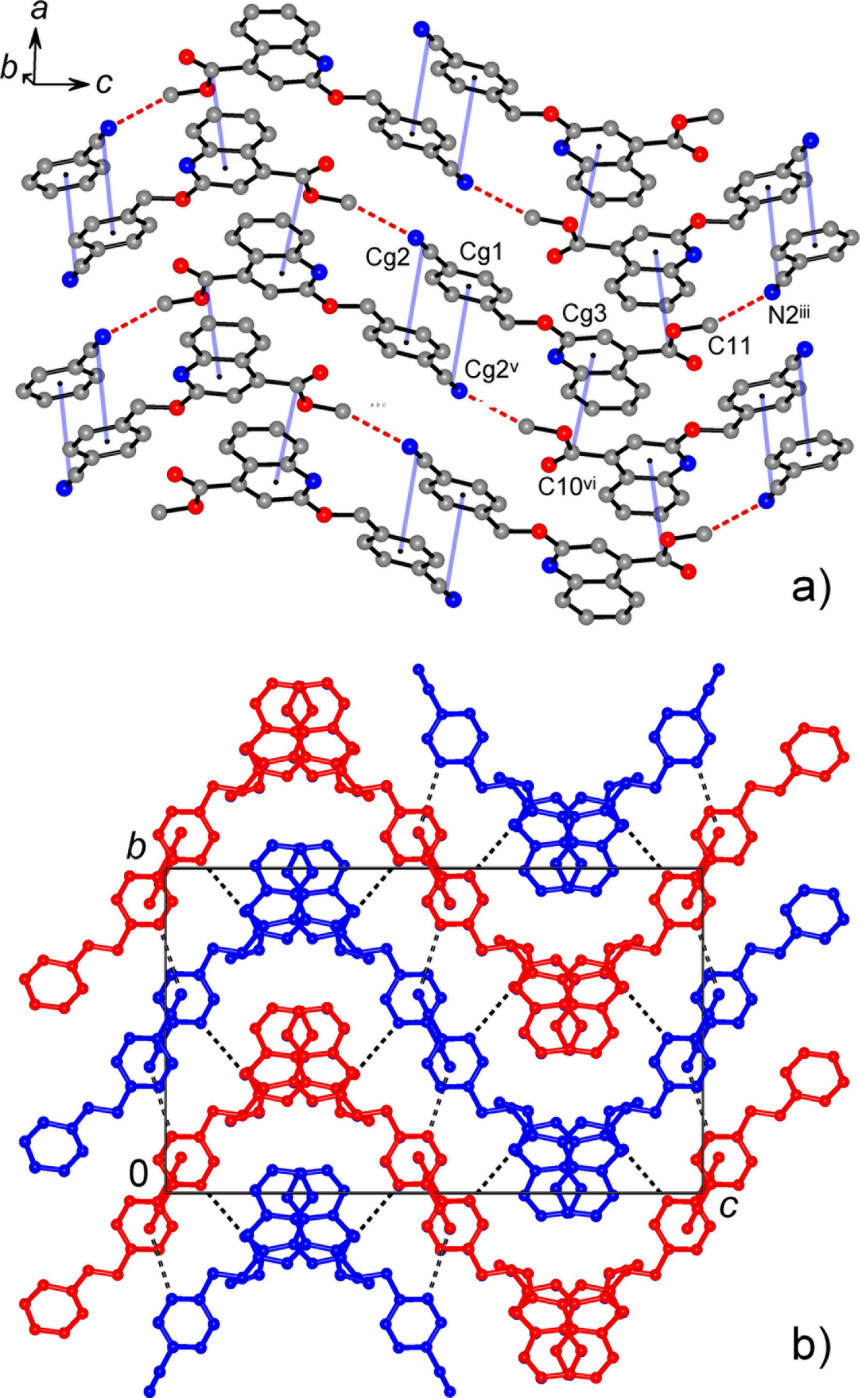
(*a*) Projection of the structure nearly on the *ac*-plane showing assembly of the layers by means of stacking inter­actions (indicated in blue) and tetrel bonds of the type OCH_3_⋯N≡C (indicated with dotted red lines). (*b*) Packing of successive corrugated layers viewed in a projection on the *bc*-plane, with dotted lines representing inter­layer weak hydrogen bonding. The individual layers are identified with blue and red colors. *Cg*1, *Cg*2 and *Cg*3 are centroids of the groups C13–C18, N2/C19 and N1/C1/C6–C9, respectively. [Symmetry codes: (iii) −*x* + 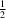, *y* − 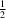, *z*; (v) −*x* + 1, −*y* + 1, −*z* + 1; (vi) *x* + 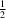, *y*, −*z* + 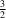.]

In the crystal, inter­molecular C15—H15⋯O1^ii^ hydrogen bonds [symmetry code (ii): *x* + 1, *y* + 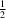, *z* + 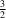; Table 1 ▸] link the mol­ecules into the infinite chains along the *b*-axis direction (Fig. 2 ▸). However, the entire non-covalent framework in the structure may be best described as consisting of corrugated layers, which propagate parallel to the *ac* plane and are linked in the third dimension by a set of very weak hydrogen bonds. The layers themselves are sustained by two kinds of stacking inter­actions. First, two inversion-related cyano­phenyl moieties [symmetry code: (v) −*x* + 1, −*y* + 1, −*z* + 1] afford anti­parallel stacks with inter­planar distances of 3.660 (2) Å, in which the centroids of C19–N2 groups [*Cg*2] are situated almost exactly above the centroids of the corresponding aromatic rings (*Cg*1) at 3.735 (2) Å (Figs. 2 ▸, 3 ▸). The second kind of stacking inter­action is identified between nearly parallel ester groups and heterocyclic rings *B* [symmetry code: (vi) *x* + 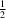, *y*, −*z* + 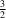; inter­planar angle is 3.98 (11)°], with separation *Cg*3⋯C10^vi^ = 3.817 (2) Å (*Cg*3 is the ring *B* centroid). These layers are further consolidated by relatively short tetrel bonding (Varadwaj *et al.*, 2023 ▸) of the type OCH_3_⋯N≡C [C11⋯N2^iv^ = 3.146 (3) Å, symmetry code (iv): −*x* + 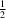, −*y* + 1, *x* + 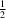], which is well compatible to both stacking patterns (Fig. 3 ▸).

The resulting corrugated layers are separated by 7.849 Å, which is a half of the unit cell parameter *b* (Fig. 3 ▸). In addition to the above most prominent C15—H15⋯O1^ii^ hydrogen bonds, the suite of inter­layer inter­actions also comprises weaker C3—H3⋯O3^i^ and C18—H18⋯N2^iii^ bonds [symmetry codes (i): *x* + 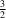, *y* − 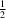, *z*; (iii) *x* + 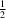, *y* − 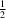, *z*; Table 1 ▸]. These inter­actions are also directional, with corresponding angles at the H atoms of 163 and 158°, respectively. No C—H⋯(ring) or π(ring)–(ring) inter­actions are observed. The title compound highlights rather the inter­play of different kinds of stacking inter­actions, weak hydrogen and tetrel bonding for consolidating the 3D architecture. The combination of Hirshfeld surface analysis and energy framework calculations reveals dispersion energy as the dominant contributor, offering new insights into the packing features of quinoline-based systems and their potential in crystal engineering.

## 4. Hirshfeld surface analysis

**Figure 4:**
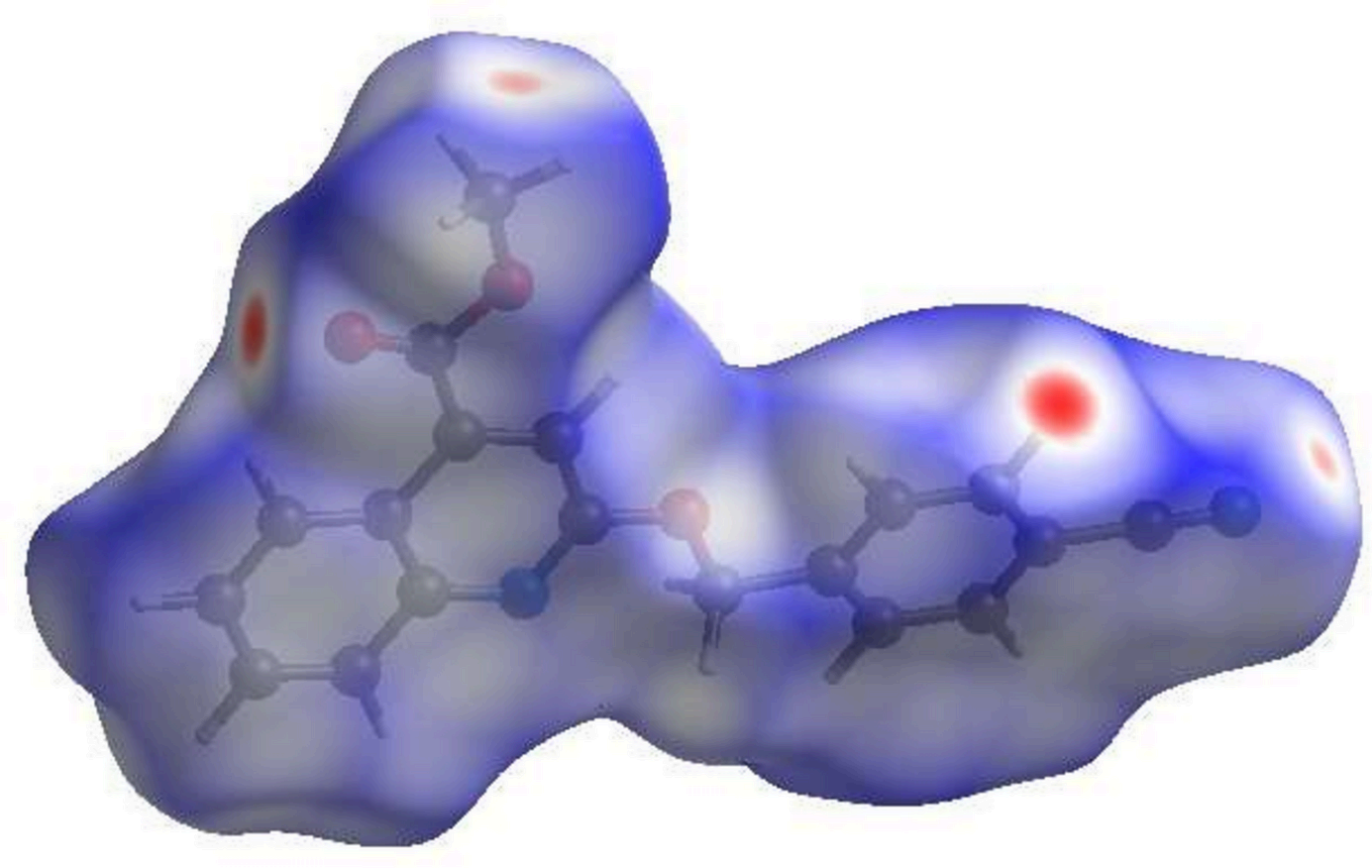
The Hirshfeld surface of the title compound mapped over *d*_norm_.

For visualizing the inter­molecular inter­actions in the crystal of the title compound, a Hirshfeld surface (HS) analysis (Hirshfeld, 1977 ▸; Spackman & Jayatilaka, 2009 ▸) was carried out using *Crystal Explorer 17.5* (Spackman *et al.*, 2021 ▸). In the HS plotted over *d*_norm_ (Fig. 4 ▸), the contact distances equal, shorter and longer than the sum of van der Waals radii are shown in white, red and blue, respectively (Venkatesan *et al.*, 2016 ▸). The brightest red spots correspond to the donor and acceptor sites of the C15—H15⋯O1^ii^ bonds, whereas the positions of the tetrel OCH_3_⋯N≡C bonds are also clearly visible as a pair of more diffuse red spots.

**Figure 5:**
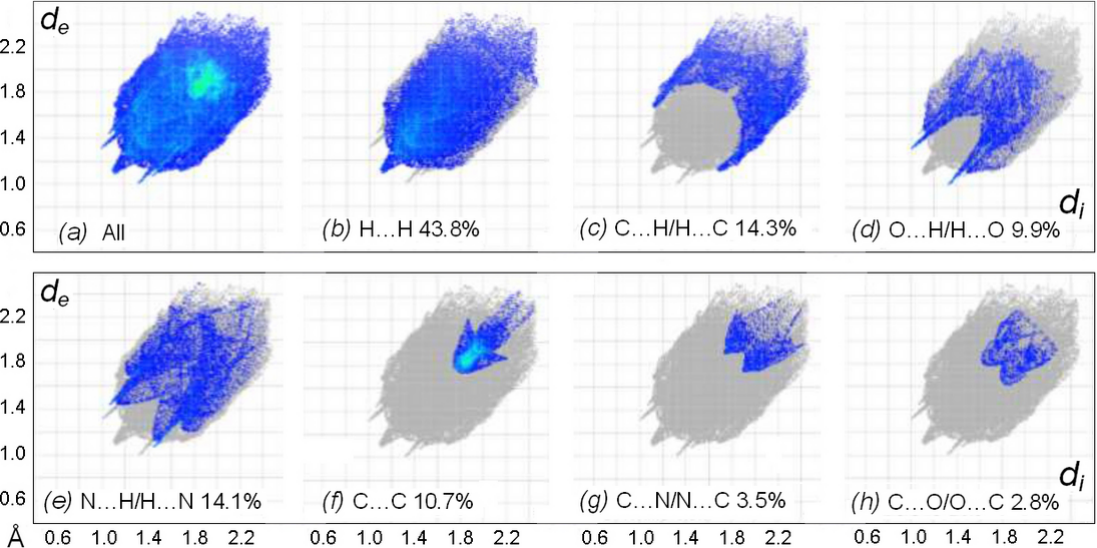
Two-dimensional fingerprint plots for the title compound: (*a*) all inter­actions and delineated into the principal contributions of (*b*) H⋯H, (*c*) C⋯H/H⋯C, (*d*) O⋯H/H⋯O, (*e*) N⋯H/H⋯N, (*f*) C⋯C, (*g*) C⋯N/N⋯C and (*h*) C⋯O/O⋯C contacts. Other minor contributors are O⋯O (0.5%) and N⋯O/O⋯N (0.4%) contacts.

The overall two-dimensional fingerprint plots and those delineated into the contributions of the individual types of the contacts (McKinnon *et al.*, 2007 ▸) are shown in Fig. 5 ▸. Beyond the expected far dominant significance of H⋯H contacts (43.8%), the main contributors to the Hirshfeld surface are also associated with the H atoms: C⋯H/H⋯C = 14.3%, N⋯H/H⋯N = 14.1% and O⋯H/H⋯O = 9.9%. However, only the latter ones appear in the plots in the form of two relatively sharp spikes pointing to the lower left, thus indicating the hydrogen-bond inter­actions (shortest H⋯O = 2.35 Å). In the case of N⋯H/H⋯N contacts, these spikes are much shorter and diffuse, since most points originate rather in the tetrel inter­actions of methyl and cyano groups. In addition, the light-blue area centered at *ca* 3.80 Å in the plot for C⋯C contacts indicates the above stacking inter­actions. In total, the corresponding contacts, *i.e.* C⋯C, C⋯N/N⋯C and C⋯O/O⋯C, deliver as much as 17.0% to the surface area.

The nearest coordination environment of a mol­ecule can be determined from the color patches on the HS based on how close to other mol­ecules they are. The Hirshfeld surface representations of contact patches plotted onto the surface are shown for the H⋯H, H⋯C/C⋯H, H⋯ N/N⋯H, C⋯C and H⋯O/O⋯H inter­actions in Fig. S2*a–e*, respectively, in the supporting information. The Hirshfeld surface analysis confirms the importance of H-atom contacts in establishing the packing. The large number of H⋯H, H⋯C/C⋯H, H⋯N/N⋯H, C⋯C and H⋯O/O⋯H inter­actions suggest that van der Waals inter­actions and hydrogen bonding play the major roles in the crystal packing (Hathwar *et al.*, 2015 ▸).

## 5. Inter­action energy calculations and energy frameworks

The inter­molecular inter­action energies were calculated using the CE–B3LYP/6–31G(d,p) energy model available in *Crystal Explorer 17.5* (Spackman *et al.*, 2021 ▸), where a cluster of mol­ecules is generated by applying crystallographic symmetry operations with respect to a selected central mol­ecule within the radius of 3.8 Å by default (Turner *et al.*, 2014 ▸). The total inter­molecular energy (*E*_tot_) is the sum of electrostatic (*E*_ele_), polarization (*E*_pol_), dispersion (*E*_dis_) and exchange-repulsion (*E*_rep_) energies (Turner *et al.*, 2015 ▸).

**Figure 6:**
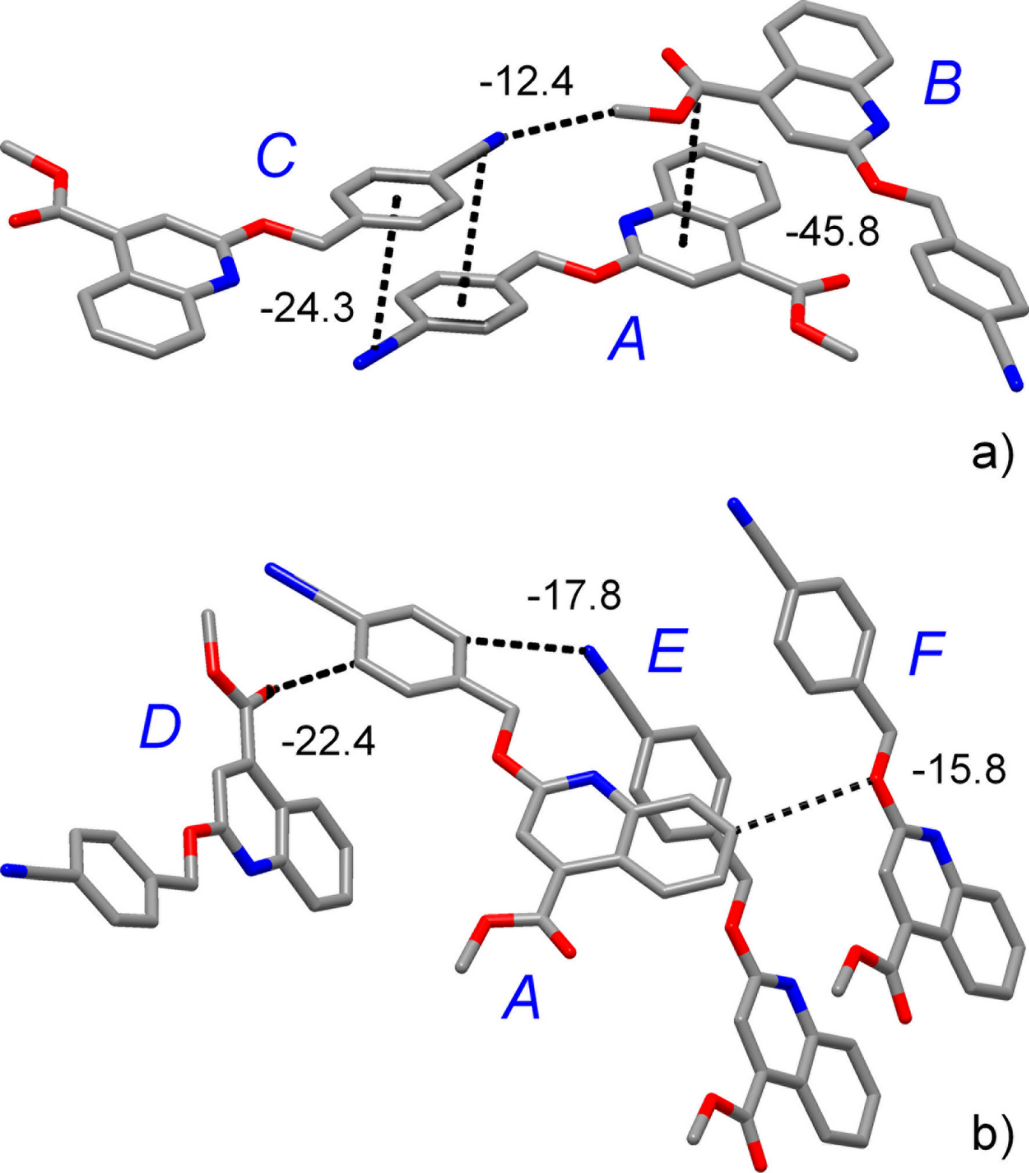
The principal pathways of inter­molecular inter­actions, identified with a cut-off limit of 12 kJ mol^−1^, which involve (*a*) stacking and tetrel inter­actions and (*a*) weak hydrogen bonding. The inter­action energies are given in kJ mol^−1^.

With a cut-off of |*E*_tot_| > 12.0 kJ mol^−1^, seven symmetry-independent paths were identified for the closest environment of the title mol­ecules (Table 2 ▸). The highest energy *E*_tot_ = −45.8 kJ mol^−1^ corresponds to the pairing pattern involving stacking of quinoline and carb­oxy groups (path *A*⋯*B*, Fig. 6 ▸). The primary contributor here is London dispersion (*E*_dis_ = −66.2 kJ mol^−1^), due to the very large inter­action area. Stacking of cyano­phenyl moieties is perceptibly weaker with *E*_tot_ = −24.3 kJ mol^−1^. This value approaches the parameter calculated for the slipped anti­parallel dimer of nitro­benzene mol­ecules (−28.2 kJ mol^−1^; Tsuzuki *et al.*, 2006 ▸). This stacking is also clearly distinguishable in the present energy landscape and it is even superior to the energies of the inter­molecular inter­actions, which correspond to weak hydrogen bonding (−15.8 and −17.8 kJ mol^−1^; Table 2 ▸). In the case of the *A*⋯*D* pair (Fig. 6 ▸), slightly higher total energy of −22.4 kJ mol^−1^ is due to a combination of weak hydrogen bond C15—H15⋯O1^ii^ and dispersion forces, with the corresponding principal contributors *E*_ele_ = −10.3 and *E*_dis_ = −25.3 kJ mol^−1^. This is in line with larger inter­action area and generation of additional vdW contacts, *e.g.* O2⋯C3^ii^ = 3.534 (2) Å. Finally, the tetrel bonds OCH_3_⋯N≡C (pair *B*⋯*C*, Fig. 6 ▸) are very similar in energy to the weak hydrogen bonds (*E*_tot_ = −12.4 kJ mol^−1^) and therefore their significance to the crystal packing may be regarded as comparable.

The evaluation of the electrostatic, dispersion and total energy frameworks indicate that the consolidation is dominated *via* the dispersion energy contributions (Fig. S2 in the supporting information).

## 6. Database survey

A search of the Cambridge Structural Database (CSD; updated 16 May 2025; Groom *et al.*, 2016 ▸) reveals 18 relevant hits, which include the 2-oxo-1,2-di­hydro­quinoline-4-carboxyl­ate core. Two of these entries, namely PEDKAO (Filali Baba *et al.*, 2022 ▸) and ROKCIG (Filali Baba *et al.*, 2019 ▸), involve additional Cl-atoms installed on the aromatic rings. Oxygen-derivatization of the selected core is a particularly rare feature. Among 13 alkyl-substituted structures retrieved, including AROPAB (Bouzian *et al.*, 2020 ▸) and SECCAH (Hayani *et al.*, 2021*b* ▸), most were identified as N-alkyl­ated derivatives. The only structural precedent for the O-alkyl­ation of the above core is provided by 2-eth­oxy-2-oxoethyl 2-(2-eth­oxy-2-oxoeth­oxy)quinoline-4-carboxyl­ate (refcode LIRKIJ; Bouzian *et al.*, 2018 ▸). This highlights the need for detailed structural validation when classifying substitution patterns on such frameworks. From a supra­molecular perspective, the crystal packing of ROKCIG reveals no π–π stacking inter­actions or C—H⋯Cl hydrogen bonds, but it differs markedly from that of the title compound. It forms an inversion dimer through C—H⋯O hydrogen bonds, lacking the chain-like hydrogen-bonded pattern seen in the title structure. In contrast, its halogen-free analog (ROKCOM; Filali Baba *et al.*, 2019 ▸) forms mol­ecular bands *via* C—H⋯O hydrogen bonding, further stabilized by weak π–π contacts.

## 7. Synthesis and crystallization

The procedure for synthesizing the methyl 2-[(4-cyano­benz­yl)­oxy]quinoline-4-carboxyl­ate derivative is as follows. To a solution of methyl 2-oxo-1,2-di­hydro­quinoline-4-carboxyl­ate (0.60 g, 2.20 mmol) in 15 ml of di­methyl­formamide (DMF), 4-(bromo­meth­yl)benzo­nitrile (0.21 ml, 2.41 mmol), K_2_CO_3_ (0.85 g, 6.10 mmol) and tetra-*n*-butyl­ammonium bromide (TBAB; 0.05 g, 0.18 mmol) were added and the reaction mixture was agitated at ambient temperature for a period of 12 h. Following completion of the reaction, the precipitated inorganic salts were removed through filtration and the solvent was evaporated under reduced pressure. The resultant residue was dissolved in di­chloro­methane. This solution was subsequently dried using anhydrous sodium sulfate and then concentrated under reduced pressure. The compound was purified through column chromatography, employing a hexa­ne/ethyl acetate eluent (4:1 *v*/*v*). The target product was obtained in a yield of 45%. It was further recrystallized from a mixture of di­chloro­methane and hexane (1:4 *v*/*v*) giving transparent colorless crystals, m.p. = 394 K. ^1^H NMR (300 MHz, CDCl_3_), δ, ppm: 8.61 (*dd*, *J* = 8.6, 1.4 Hz, 1H, CH_Ar_), 7.90–7.86 (*m*, 1H, CH_Ar_), 7.70–7.60 (*m*, 5H, CH_Ar_), 7.53–7.47 (*m*, 2H, CH_Ar_), 5.63 (*s*, 2H, CH_2_), 4.03 (*s*, 3H, CH_3_). ^13^C NMR (75 MHz, CDCl_3_), δ, ppm: 166.21, 160.57, 147.34, 142.64, 138.51, 132.40, 130.25, 128.35, 127.85, 125.77, 125.74, 122.1, 118.87, 115.18, 111.74, 66.82, 52.87. FT–IR (cm^−1^): 2858 (C—H_sp_^3^), 1727 (C=O), 2226 (C≡N), 1575–1607 (C=C, aromatic stretching); 1238 (C—O—C, ether bond).

## 8. Refinement

Crystal data, data collection and structure refinement details are summarized in Table 3 ▸. H atoms were positioned geometrically and refined as riding, with C—H = 0.95 Å (aromatic CH), 0.97 Å (CH_2_) and 0.98 Å (CH_3_) and with *U*_iso_(H) = 1.2*U*_eq_ or 1.5U_eq_ of the carrier C-atom for CH and CH_2_ or CH_3_ groups, respectively. Four outliers (108, 204, 222 and 232) were omitted in the last cycles of refinement.

## Supplementary Material

Crystal structure: contains datablock(s) I. DOI: 10.1107/S2056989025005547/nu2008sup1.cif

Structure factors: contains datablock(s) I. DOI: 10.1107/S2056989025005547/nu2008Isup2.hkl

Supporting information file. DOI: 10.1107/S2056989025005547/nu2008Isup3.cdx

Supporting information file. DOI: 10.1107/S2056989025005547/nu2008Isup4.cml

Supporting information file. DOI: 10.1107/S2056989025005547/nu2008sup5.pdf

CCDC reference: 2465703

Additional supporting information:  crystallographic information; 3D view; checkCIF report

## Figures and Tables

**Table 1:** Hydrogen-bond geometry (Å, °)

*D*—H⋯*A*	*D*—H	H⋯*A*	*D*⋯*A*	*D*—H⋯*A*
C3—H3⋯O3^i^	0.93	2.75	3.651 (2)	163
C5—H5⋯O1	0.93	2.25	2.883 (2)	125
C15—H15⋯O1^ii^	0.93	2.48	3.337 (2)	154
C18—H18⋯N2^iii^	0.93	2.68	3.558 (3)	158

Symmetry codes: (i) 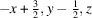; (ii) 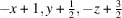; (iii) 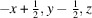.

**Table 2:** Calculated inter­action energies (kJ mol^−1^) Inter­action energies were calculated employing the CE-B3LYP/6–31G(d,p) functional/basis set combination. The scale factors used to determine *E*_tot_ are *k*_ele_ = 1.057, *k*_pol_ = 0.740, *k*_dis_ = 0.871, and *k*_rep_ = 0.618 (Mackenzie *et al.*, 2017 ▸). *R* is the distance between the centroids of the inter­acting mol­ecules.

Path	Symmetry code	Type^*a*^	*R* (Å)	*E*_ele_	*E*_pol_	*E*_dis_	*E*_rep_	*E*_tot_
*A*⋯*B*	*x* + 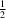 , *y*, −*z* + 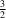	stacking	5.77	−5.3	−2.1	−66.2	30.8	−45.8
*A*⋯*C*	−*x* + 1, −*y* + 1, −*z* + 1	stacking	12.05	−6.1	−2.4	−25.1	9.3	−24.3
*B*⋯*C*	−*x* + 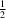 , −*y* + 1, *z* + 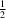	tetrel	15.89	−10.5	−2.7	−5.0	8.2	−12.4
*A*⋯*D*	*x* + 1, *y* + 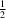 , *z* + 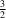	C—H⋯O, dispersion	8.93	−10.3	−2.0	−25.3	19.5	−22.4
*A*⋯*E*	−*x* + 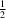 , *y* + 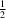 , *z*	C—H⋯N	8.73	−10.1	−2.7	−11.7	8.2	−17.8
*A*⋯*F*	*x* + 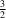 , *y* − 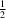 , *z*	C—H⋯O	8.79	−4.2	−0.5	−20.4	10.8	−15.8
*A*⋯*G*	*x* + 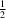 , −*y* + 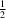 , −*z* + 1	dispersion	9.56	−4.3	−1.6	−16.0	7.4	−15.1

Note: (*a*) For details of the inter­action modes see Fig. 6 ▸. Weak dispersion inter­action *A*⋯*G* is not shown in the Figure.

**Table 3:** Experimental details

Crystal data	
Chemical formula	C_19_H_14_N_2_O_3_
*M*_r_	318.32
Crystal system, space group	Orthorhombic, *Pbca*
Temperature (K)	299
*a*, *b*, *c* (Å)	7.7810 (6), 15.6978 (11), 25.966 (2)
*V* (Å^3^)	3171.6 (4)
*Z*	8
Radiation type	Mo *K*α
μ (mm^−1^)	0.09
Crystal size (mm)	0.26 × 0.22 × 0.19

Data collection	
Diffractometer	Bruker D8 VENTURE PHOTON 3 CPAD
Absorption correction	Multi-scan (*SADABS*; Krause *et al.*, 2015 ▸)
*T*_min_, *T*_max_	0.719, 0.745
No. of measured, independent and observed [*I* > 2σ(*I*)] reflections	78015, 3234, 2897
*R*_int_	0.041
(sin θ/λ)_max_ (Å^−1^)	0.625

Refinement	
*R*[*F*^2^ > 2σ(*F*^2^)], *wR*(*F*^2^), *S*	0.049, 0.136, 1.05
No. of reflections	3234
No. of parameters	218
H-atom treatment	H-atom parameters constrained
Δρ_max_, Δρ_min_ (e Å^−3^)	0.25, −0.21

Computer programs: *APEX4* (Bruker, 2019 ▸), *SAINT* (Bruker, 2016 ▸), *SHELXT2018/2* (Sheldrick, 2015*a* ▸), *SHELXL2019/1* (Sheldrick, 2015*b* ▸), *DIAMOND* (Brandenburg, 1999 ▸) and *WinGX* (Farrugia, 2012 ▸).

## supplementary crystallographic information

### Methyl 2-[(4-cyanophenyl)methoxy]quinoline-4-carboxylate Crystal data

**Table:**

C_19_H_14_N_2_O_3_	*D*_x_ = 1.333 Mg m^−^^3^
*M**_r_* = 318.32	Mo *K*α radiation, λ = 0.71073 Å
Orthorhombic, *P**b**c**a*	Cell parameters from 9947 reflections
*a* = 7.7810 (6) Å	θ = 3.0–26.1°
*b* = 15.6978 (11) Å	µ = 0.09 mm^−^^1^
*c* = 25.966 (2) Å	*T* = 299 K
*V* = 3171.6 (4) Å^3^	Prism, colourless
*Z* = 8	0.26 × 0.22 × 0.19 mm
*F*(000) = 1328	

### Methyl 2-[(4-cyanophenyl)methoxy]quinoline-4-carboxylate Data collection

**Table:**

Bruker D8 VENTURE PHOTON 3 CPAD diffractometer	2897 reflections with *I* > 2σ(*I*)
Radiation source: microfocus sealed X-ray tube	*R*_int_ = 0.041
φ and ω scans	θ_max_ = 26.4°, θ_min_ = 2.6°
Absorption correction: multi-scan (SADABS; Krause *et al.*, 2015)	*h* = −9→9
*T*_min_ = 0.719, *T*_max_ = 0.745	*k* = −19→17
78015 measured reflections	*l* = −32→32
3234 independent reflections	

### Methyl 2-[(4-cyanophenyl)methoxy]quinoline-4-carboxylate Refinement

**Table:**

Refinement on *F*^2^	Primary atom site location: structure-invariant direct methods
Least-squares matrix: full	Secondary atom site location: difference Fourier map
*R*[*F*^2^ > 2σ(*F*^2^)] = 0.049	Hydrogen site location: inferred from neighbouring sites
*wR*(*F*^2^) = 0.136	H-atom parameters constrained
*S* = 1.05	*w* = 1/[σ^2^(*F*_o_^2^) + (0.0634*P*)^2^ + 1.2076*P*] where *P* = (*F*_o_^2^ + 2*F*_c_^2^)/3
3234 reflections	(Δ/σ)_max_ < 0.001
218 parameters	Δρ_max_ = 0.25 e Å^−^^3^
0 restraints	Δρ_min_ = −0.21 e Å^−^^3^

### Methyl 2-[(4-cyanophenyl)methoxy]quinoline-4-carboxylate Special details

**Table:**

Geometry. All esds (except the esd in the dihedral angle between two l.s. planes) are estimated using the full covariance matrix. The cell esds are taken into account individually in the estimation of esds in distances, angles and torsion angles; correlations between esds in cell parameters are only used when they are defined by crystal symmetry. An approximate (isotropic) treatment of cell esds is used for estimating esds involving l.s. planes.

### Methyl 2-[(4-cyanophenyl)methoxy]quinoline-4-carboxylate Fractional atomic coordinates and isotropic or equivalent isotropic displacement parameters (Å^2^)

**Table:**

	*x*	*y*	*z*	*U*_iso_*/*U*_eq_	
O1	0.6069 (3)	0.13417 (10)	0.85140 (5)	0.1081 (8)	
O2	0.4881 (2)	0.25179 (9)	0.82571 (5)	0.0777 (5)	
O3	0.46253 (17)	0.26146 (7)	0.64306 (4)	0.0539 (3)	
N1	0.58631 (19)	0.12898 (8)	0.65387 (5)	0.0472 (3)	
N2	0.1671 (3)	0.61040 (12)	0.47079 (7)	0.0908 (7)	
C1	0.6466 (2)	0.06874 (9)	0.68811 (6)	0.0434 (4)	
C2	0.7132 (3)	−0.00718 (11)	0.66761 (7)	0.0587 (5)	
H2	0.7149	−0.0151	0.6321	0.070*	
C3	0.7751 (3)	−0.06941 (11)	0.69907 (8)	0.0626 (5)	
H3	0.8188	−0.1193	0.6849	0.075*	
C4	0.7735 (3)	−0.05892 (11)	0.75228 (8)	0.0573 (4)	
H4	0.8159	−0.1018	0.7734	0.069*	
C5	0.7101 (2)	0.01379 (10)	0.77346 (6)	0.0490 (4)	
H5	0.7105	0.0202	0.8091	0.059*	
C6	0.64375 (19)	0.07983 (9)	0.74226 (6)	0.0394 (3)	
C7	0.57211 (19)	0.15814 (9)	0.76106 (5)	0.0379 (3)	
C8	0.5129 (2)	0.21656 (9)	0.72667 (5)	0.0407 (3)	
H8	0.4662	0.2678	0.7380	0.049*	
C9	0.5236 (2)	0.19824 (9)	0.67342 (6)	0.0420 (4)	
C10	0.5590 (2)	0.17753 (10)	0.81731 (6)	0.0459 (4)	
C11	0.4652 (3)	0.27757 (17)	0.87850 (7)	0.0821 (7)	
H11A	0.4454	0.3379	0.8799	0.123*	
H11B	0.5666	0.2638	0.8979	0.123*	
H11C	0.3683	0.2482	0.8929	0.123*	
C12	0.4609 (3)	0.24802 (11)	0.58871 (6)	0.0529 (4)	
H12A	0.3873	0.2002	0.5802	0.063*	
H12B	0.5761	0.2357	0.5765	0.063*	
C13	0.3942 (2)	0.32785 (10)	0.56377 (6)	0.0455 (4)	
C14	0.3756 (3)	0.40331 (11)	0.59018 (6)	0.0630 (5)	
H14	0.4035	0.4054	0.6250	0.076*	
C15	0.3163 (3)	0.47576 (11)	0.56604 (7)	0.0668 (6)	
H15	0.3036	0.5261	0.5845	0.080*	
C16	0.2760 (3)	0.47327 (10)	0.51442 (6)	0.0531 (4)	
C17	0.2941 (3)	0.39821 (11)	0.48727 (7)	0.0680 (6)	
H17	0.2664	0.3962	0.4525	0.082*	
C18	0.3534 (3)	0.32634 (11)	0.51194 (6)	0.0630 (5)	
H18	0.3663	0.2760	0.4935	0.076*	
C19	0.2146 (3)	0.54952 (12)	0.48955 (7)	0.0663 (6)	

### Methyl 2-[(4-cyanophenyl)methoxy]quinoline-4-carboxylate Atomic displacement parameters (Å^2^)

**Table:**

	*U*^11^	*U*^22^	*U*^33^	*U*^12^	*U*^13^	*U*^23^
O1	0.207 (2)	0.0747 (10)	0.0423 (7)	0.0505 (12)	−0.0176 (10)	0.0059 (7)
O2	0.1235 (13)	0.0744 (9)	0.0352 (6)	0.0408 (9)	0.0013 (7)	−0.0046 (6)
O3	0.0886 (9)	0.0396 (6)	0.0336 (6)	0.0134 (6)	−0.0052 (6)	0.0023 (4)
N1	0.0644 (8)	0.0390 (7)	0.0384 (7)	0.0040 (6)	−0.0002 (6)	0.0014 (5)
N2	0.148 (2)	0.0554 (10)	0.0691 (11)	0.0207 (11)	−0.0237 (12)	0.0127 (9)
C1	0.0493 (8)	0.0364 (7)	0.0445 (8)	0.0001 (6)	0.0009 (7)	0.0025 (6)
C2	0.0774 (12)	0.0459 (9)	0.0529 (10)	0.0109 (9)	0.0050 (9)	−0.0033 (7)
C3	0.0752 (13)	0.0415 (9)	0.0710 (12)	0.0152 (9)	0.0028 (10)	−0.0019 (8)
C4	0.0602 (10)	0.0420 (9)	0.0697 (11)	0.0072 (8)	−0.0049 (9)	0.0116 (8)
C5	0.0531 (9)	0.0437 (8)	0.0501 (9)	−0.0003 (7)	−0.0043 (7)	0.0093 (7)
C6	0.0395 (8)	0.0354 (7)	0.0433 (8)	−0.0045 (6)	−0.0002 (6)	0.0042 (6)
C7	0.0398 (7)	0.0367 (7)	0.0373 (7)	−0.0051 (6)	−0.0001 (6)	0.0037 (6)
C8	0.0505 (9)	0.0338 (7)	0.0376 (8)	0.0005 (6)	0.0011 (6)	0.0007 (6)
C9	0.0530 (9)	0.0353 (7)	0.0376 (8)	0.0009 (6)	−0.0015 (6)	0.0042 (6)
C10	0.0553 (9)	0.0444 (8)	0.0380 (8)	−0.0031 (7)	−0.0015 (7)	0.0048 (6)
C11	0.1124 (18)	0.0963 (16)	0.0377 (10)	0.0283 (14)	0.0028 (11)	−0.0135 (10)
C12	0.0792 (12)	0.0454 (8)	0.0341 (8)	0.0106 (8)	−0.0042 (8)	−0.0011 (6)
C13	0.0622 (10)	0.0387 (8)	0.0357 (7)	0.0012 (7)	−0.0037 (7)	0.0008 (6)
C14	0.1068 (16)	0.0476 (9)	0.0347 (8)	0.0127 (10)	−0.0189 (9)	−0.0046 (7)
C15	0.1149 (17)	0.0423 (9)	0.0432 (9)	0.0134 (10)	−0.0172 (10)	−0.0075 (7)
C16	0.0788 (12)	0.0389 (8)	0.0416 (8)	0.0011 (8)	−0.0119 (8)	0.0033 (6)
C17	0.1211 (18)	0.0473 (9)	0.0356 (8)	0.0025 (10)	−0.0210 (10)	0.0000 (7)
C18	0.1131 (16)	0.0393 (8)	0.0366 (8)	0.0046 (9)	−0.0112 (9)	−0.0049 (7)
C19	0.1043 (16)	0.0469 (10)	0.0476 (9)	0.0043 (10)	−0.0163 (10)	0.0023 (8)

### Methyl 2-[(4-cyanophenyl)methoxy]quinoline-4-carboxylate Geometric parameters (Å, º)

**Table:**

O1—C10	1.177 (2)	C7—C10	1.496 (2)
O2—C10	1.308 (2)	C8—C9	1.415 (2)
O2—C11	1.440 (2)	C8—H8	0.9300
O3—C9	1.3535 (18)	C11—H11A	0.9600
O3—C12	1.4269 (18)	C11—H11B	0.9600
N1—C9	1.296 (2)	C11—H11C	0.9600
N1—C1	1.380 (2)	C12—C13	1.503 (2)
N2—C19	1.135 (2)	C12—H12A	0.9700
C1—C2	1.404 (2)	C12—H12B	0.9700
C1—C6	1.417 (2)	C13—C14	1.376 (2)
C2—C3	1.362 (2)	C13—C18	1.383 (2)
C2—H2	0.9300	C14—C15	1.378 (2)
C3—C4	1.391 (3)	C14—H14	0.9300
C3—H3	0.9300	C15—C16	1.377 (2)
C4—C5	1.360 (2)	C15—H15	0.9300
C4—H4	0.9300	C16—C17	1.380 (2)
C5—C6	1.413 (2)	C16—C19	1.442 (2)
C5—H5	0.9300	C17—C18	1.377 (2)
C6—C7	1.435 (2)	C17—H17	0.9300
C7—C8	1.360 (2)	C18—H18	0.9300
			
C10—O2—C11	117.47 (15)	O2—C10—C7	111.91 (13)
C9—O3—C12	118.09 (12)	O2—C11—H11A	109.5
C9—N1—C1	116.79 (13)	O2—C11—H11B	109.5
N1—C1—C2	117.56 (14)	H11A—C11—H11B	109.5
N1—C1—C6	123.34 (14)	O2—C11—H11C	109.5
C2—C1—C6	119.09 (14)	H11A—C11—H11C	109.5
C3—C2—C1	120.79 (16)	H11B—C11—H11C	109.5
C3—C2—H2	119.6	O3—C12—C13	107.82 (13)
C1—C2—H2	119.6	O3—C12—H12A	110.1
C2—C3—C4	120.51 (16)	C13—C12—H12A	110.1
C2—C3—H3	119.7	O3—C12—H12B	110.1
C4—C3—H3	119.7	C13—C12—H12B	110.1
C5—C4—C3	120.29 (16)	H12A—C12—H12B	108.5
C5—C4—H4	119.9	C14—C13—C18	118.39 (15)
C3—C4—H4	119.9	C14—C13—C12	122.62 (14)
C4—C5—C6	121.09 (16)	C18—C13—C12	118.97 (14)
C4—C5—H5	119.5	C13—C14—C15	121.25 (15)
C6—C5—H5	119.5	C13—C14—H14	119.4
C5—C6—C1	118.22 (14)	C15—C14—H14	119.4
C5—C6—C7	125.11 (14)	C16—C15—C14	119.71 (16)
C1—C6—C7	116.66 (13)	C16—C15—H15	120.1
C8—C7—C6	119.06 (13)	C14—C15—H15	120.1
C8—C7—C10	118.73 (13)	C15—C16—C17	119.87 (15)
C6—C7—C10	122.21 (13)	C15—C16—C19	119.21 (15)
C7—C8—C9	118.99 (14)	C17—C16—C19	120.92 (14)
C7—C8—H8	120.5	C18—C17—C16	119.73 (15)
C9—C8—H8	120.5	C18—C17—H17	120.1
N1—C9—O3	121.28 (14)	C16—C17—H17	120.1
N1—C9—C8	125.15 (14)	C17—C18—C13	121.04 (15)
O3—C9—C8	113.57 (13)	C17—C18—H18	119.5
O1—C10—O2	121.57 (16)	C13—C18—H18	119.5
O1—C10—C7	126.52 (16)	N2—C19—C16	178.7 (2)
			
C9—N1—C1—C2	179.24 (16)	C12—O3—C9—C8	−177.52 (15)
C9—N1—C1—C6	−0.5 (2)	C7—C8—C9—N1	0.0 (3)
N1—C1—C2—C3	179.93 (18)	C7—C8—C9—O3	−179.29 (14)
C6—C1—C2—C3	−0.3 (3)	C11—O2—C10—O1	−2.1 (3)
C1—C2—C3—C4	0.1 (3)	C11—O2—C10—C7	178.98 (18)
C2—C3—C4—C5	−0.1 (3)	C8—C7—C10—O1	−178.7 (2)
C3—C4—C5—C6	0.4 (3)	C6—C7—C10—O1	1.8 (3)
C4—C5—C6—C1	−0.7 (2)	C8—C7—C10—O2	0.2 (2)
C4—C5—C6—C7	179.06 (16)	C6—C7—C10—O2	−179.31 (15)
N1—C1—C6—C5	−179.64 (15)	C9—O3—C12—C13	−177.94 (14)
C2—C1—C6—C5	0.6 (2)	O3—C12—C13—C14	12.1 (3)
N1—C1—C6—C7	0.6 (2)	O3—C12—C13—C18	−169.42 (18)
C2—C1—C6—C7	−179.15 (15)	C18—C13—C14—C15	0.6 (3)
C5—C6—C7—C8	179.86 (15)	C12—C13—C14—C15	179.1 (2)
C1—C6—C7—C8	−0.4 (2)	C13—C14—C15—C16	−0.5 (4)
C5—C6—C7—C10	−0.6 (2)	C14—C15—C16—C17	0.4 (4)
C1—C6—C7—C10	179.15 (14)	C14—C15—C16—C19	−179.9 (2)
C6—C7—C8—C9	0.1 (2)	C15—C16—C17—C18	−0.4 (4)
C10—C7—C8—C9	−179.43 (14)	C19—C16—C17—C18	179.9 (2)
C1—N1—C9—O3	179.42 (14)	C16—C17—C18—C13	0.5 (4)
C1—N1—C9—C8	0.2 (2)	C14—C13—C18—C17	−0.6 (3)
C12—O3—C9—N1	3.2 (2)	C12—C13—C18—C17	−179.1 (2)

### Methyl 2-[(4-cyanophenyl)methoxy]quinoline-4-carboxylate Hydrogen-bond geometry (Å, º)

**Table:**

*D*—H···*A*	*D*—H	H···*A*	*D*···*A*	*D*—H···*A*
C3—H3···O3^i^	0.93	2.75	3.651 (2)	163
C5—H5···O1	0.93	2.25	2.883 (2)	125
C15—H15···O1^ii^	0.93	2.48	3.337 (2)	154
C18—H18···N2^iii^	0.93	2.68	3.558 (3)	158

Symmetry codes: (i) −*x*+3/2, *y*−1/2, *z*; (ii) −*x*+1, *y*+1/2, −*z*+3/2; (iii) −*x*+1/2, *y*−1/2, *z*.
