# Supplementary material for: Crystal structure and Hirshfeld surface analyses, inter­action energy calculations and energy frameworks of methyl 2-[(4-cyano­phen­yl)meth­oxy]quinoline-4-carboxyl­ate
Source: Acta Crystallogr E Crystallogr Commun. 2025 Jun 27;81(Pt 7):650–6. doi: 10.1107/S2056989025005547 (PMC12230613; doi:10.1107/S2056989025005547)
Supplement: Supplementary file 5 [file e-81-00650-sup5.pdf]

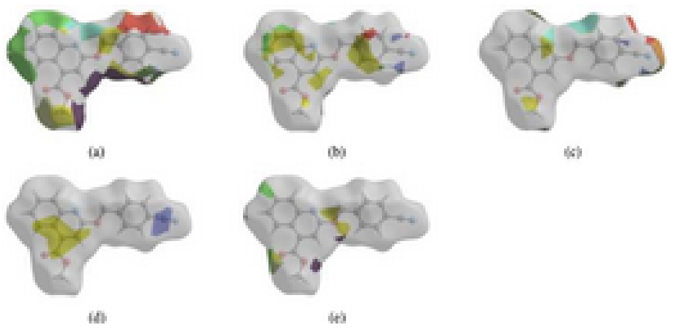

Fig. S1. The Hirshfeld surface representations of contact patches plotted onto the surface for (*a*) H···H, (*b*) H···C/C···H, (*c*) H···N/N···H, (*d*) C···C and (*e*) H···O/O···H interactions.

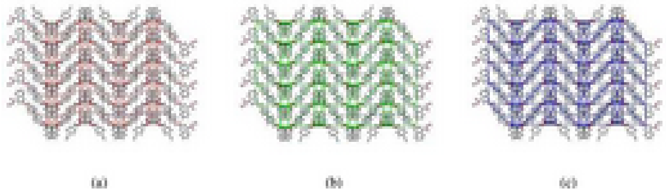

Fig. S2.
The energy frameworks for a cluster of molecules of the title compound viewed down the *a*-axis direction showing the (*a*) electrostatic energy, (*b*) dispersion energy and (*c*) total energy diagrams. The cylindrical radius is proportional to the relative strength of the corresponding energies and they were adjusted to the same scale factor of 80 with cut-off value of 5 kJ/mol within 2×2×2 unit cells.
